# An important component to investigating STEM persistence: the development and validation of the science identity (SciID) scale

**DOI:** 10.1186/s40594-022-00351-1

**Published:** 2022-05-02

**Authors:** Mary Elizabeth Lockhart, Oi-Man Kwok, Myeongsun Yoon, Raymond Wong

**Affiliations:** 1 Department of Teaching, Leaning and Culture, Texas A&M University, 4232 TAMU, College Station, TX 77843 USA; 2 Department of Educational Psychology, Texas A&M University, 4225 TAMU, College Station, TX 77843 USA; 3 Department of Statistics, Texas A&M University, 3143 TAMU, College Station, TX 77843 USA

**Keywords:** STEM education, Science identity, High school students, Instrument, Measure, Validation

## Abstract

**Background:**

Science, technology, engineering, and mathematics (STEM) influence almost every aspect of our daily lives. However, despite the high demand for STEM occupational talent, the STEM pipeline continues leaking, with less than one-sixth of high school students pursuing STEM majors and only 50% of entering STEM college majors matriculating into STEM fields. *Science identity* has been identified as the most powerful predictor of high school students pursuing an undergraduate STEM major as reported by Chang (Machine learning approach to predicting STEM college major choice, American Educational Research Association (AERA), San Francisco, 2020). Though the construct is gaining lots of attention, it remains largely ill-defined, not operationalized at the high school level, and not based upon traditional identity theory. The purpose of this study was to develop a valid and reliable instrument that measures high school students’ science identity, the Science Identity (SciID) Scale.

**Results:**

Subject experts and a small group of high school students provided content validation for the proposed scale. Exploratory factor analysis revealed an optimal two-factor solution, reflecting the traditional two-dimensions of identity theory: Exploration and Commitment. Cronbach’s alpha revealed good internal consistency for both factors. Finally, structural equation modeling confirmed the convergent validity of the instrument with the external variables of science achievement and science career interest. Furthermore, the divergent validity between science identity and science self-concept was also confirmed.

**Conclusions:**

Initial results indicate that the SciID Scale is a valid and reliable instrument that accurately measures a high school student’s standing on this construct. The soundness of this instrument will enable policy makers and practitioners to design more effective intervention programs aimed at cultivating high school students’ science identity.

## Introduction

For two decades, the United States of America has struggled to meet the call for reform set forth by the Glenn Commission claiming that we have yet to capture the attention of our students in science, technology, engineering, and mathematics (STEM) (National Commission on Mathematics and Science, 2000). Practitioners have sought to answer this call by instituting programmatic and curricular changes within STEM education. In addition, the federal government has poured money into the STEM initiative, increasing STEM research funds in inflation-adjusted dollars by over 33% from 2000 to 2011, with over $3 billion allocated annually to STEM education programs (Science and Engineering Indicators Digest, 2014). Alarmingly, the STEM pipeline continues to leak, with less than one-sixth of high school students pursuing STEM majors and only 50% of entering STEM college majors matriculating into STEM fields (U.S. Department of Education, 2015).

Part of the methodology employed in recent years in measuring the effectiveness of STEM interventions at increasing STEM persistence has been geared towards documenting changes in students’ science identities. Several studies have found that identification with context-relevant identities such as “student” or “scientist” provides a better prediction of academic performance and persistence than either racial or ethnic identity (Bonous-Hammarth, 2000; Chemers et al., 2011; Eccles & Barber, 1999; Osborne & Walker, 2006). As noted in Hazari et al. (2018), science identity-based frameworks have proven fruitful in studying science persistence with several studies showing that science identity influences science persistence. Recently, Chang and colleagues (2020) applied the machine learning approach to a large-scale national data set of high school students, the High School Longitudinal Study of 2009 (National Center for Education Statistics, 2015), and similarly found that the students’ “science identity” was the single-best predictor of their pursuit of STEM majors.

The notion of science identity as the greatest predictor of STEM persistence holds extreme consequences for the future. That is, if STEM educational interventions effectively cultivate students’ science identities, an increase in matriculation into STEM majors and careers should subsequently result. However, this particular identity domain remains ill-defined, not operationalized at the high school level, and not based upon traditional identity theory as revealed by a systematic literature review of science identity instruments (Lockhart, 2021). No instrument was created for the purpose of specifically measuring science identity within high school students, and many existing instruments have often equated science identity with a student’s science self-concept (e.g., science “kind of person”). Certainly, a brief one or two-item measure of science self-concept could reflect a portion of a student’s science identity and be useful for researchers who need a quick measure of one’s science identity. Nevertheless, the number of items in a measure is a function of reliability (Raykov & Marcoulides, 2011). Thus, a single-item is generally not the ideal way to measure educational or psychological related constructs. Furthermore, equating science self-concept and science identity hinders our understanding of the more extensive nature of the science identity construct. This equating also demonstrates a distinctive break from traditional identity theory initiated by Erikson (1959, 1968) and operationalized by Marcia (1966). Hence, as science identity is currently gaining traction and has been shown to be the greatest predictor of STEM persistence for high school students, an urgent need exists for a more traditional, theoretically grounded and psychometrically sound science identity measure for high school students who are at the critical junction of determining their educational future.

To fill this gap in the literature, in this study, we aimed to develop a new science identity measure specifically for high school students following Crocker and Algina’s (2008) recommendations for instrument development. The following research questions were addressed in the development of this scale:

1. What is the dimension (latent structure) of science identity?
2. Is the newly developed Science Identity (SciID) Scale a valid and reliable instrument?

### Review of existing literature

A thorough literature review was conducted to investigate the constructs of identity, academic identity, and science identity. Identity theory was first initiated by Erikson and operationalized by Marcia. Erickson (1959, 1968) believed that identity was a primary task of adolescence, resulting from individuals beginning to cope with social and developmental demands while seeking to provide meaning to their life choices and commitments (Bosma & Kunnen, 2008; Hewlett, 2013; Jensen, 2011; McLean & Syed, 2014; Schwartz et al., 2011; Was et al., 2009). Thus, adolescents must make important decisions in multiple identity domains, such as education and interpersonal relationships, that lead to identity synthesis or crisis (Albarello et al., 2017; Branje et al., 2014; McLean et al., 2016). Marcia (1966) operationalized Erikson’s theory, postulating that identity formation is based on two successive identity processes, Exploration and Commitment (Piotrowski, 2018).

Exploration (or Crisis) was defined by Marcia (1966) as being a “period of engagement in choosing amongst meaningful alternatives” (p. 551). The second process, Commitment, was defined by Marcia (1966) as “the degree of personal investment the individual exhibits” (p. 551).

Marcia (1966) further crossed these two identity processes with respect to their level of presence or absence within an individual and developed a series of four identity statuses: Achievement, Foreclosure, Moratorium, and Diffusion*.* Marcia’s theory has subsequently been applied to various identity domains in an effort to accurately assign individuals to an identity status (Crocetti et al., 2008a, 2008b, 2012; Marcia, 1966; Meeus, 2011; Meeus et al., 2010; Rahiminezhad et al., 2011; Was et al., 2009). Other derivations of identity theory have been developed and utilized for generations, such as social identity theory in sociology (Tajfel & Turner, 1979). However, the distinct benefit of traditional identity theory established by Erickson (1959) and Marcia (1966) is this unique ability to assign individuals into different identity statuses across various domains so as to gain insight into their identity development, trajectory and stability over time and across domains. An identity instrument’s reflection of both the Exploration and Commitment dimensions is critical for the establishment of these identity statuses.

Researchers have expanded upon Erickson and Marcia’s two-dimensional model. Luyckx and colleagues (2008) in their dual-cycle model expanded the traditional Exploration and Commitment model into five identity processes, depicting how identity commitments are formed, evaluated, and maintained. Crocetti and colleagues (2008b) incorporated Luyckx et al.’s (2006) work regarding the duality of exploration (exploration in-breadth and exploration in-depth), yet primarily focused on the specific identity processes of forming and evaluating one’s commitments. The Meeus–Crocetti Model (Crocetti et al., 2008b) proposed three dimensions to identity formation and maintenance: commitment (processes of consigning oneself to particular identity choices), exploration in-depth (a process of conscious monitoring of commitments), and reconsideration of commitments (deciding whether present commitments need to be changed).

Though these works have been impactful and utilized in research regarding identity formation and maintenance within high school students (see Porfeli et al., 2011), they were not chosen for the foundation of this particular study. A distinction is identifiable between these expanded models and Erickson and Marcia’s original work. Indeed, these newer models include a distinctive focus on a maintenance of identity, specifically of commitments, and not simply its formation. Furthermore, evidence of identity statuses arising from the proposed dimensions is somewhat disjointed. The Meeus–Crocetti Model, for example, proposed five identity statuses arising from their three proposed identity dimensions (Crocetti et al., 2008a). A total of eight (2^3^) statuses, however, are mathematically possible and yet the feasibility of the additional three statuses was not stated or explored (Crocetti et al., 2008a). Thus, Erickson and Marcia’s original model focusing on identity formation within adolescents in conjunction with the definitions of Exploration (or exploration in-breadth) and Commitment agreed upon by various authors (Crocetti et al., 2008b; Luyckx, 2006; Marcia, 1966) was selected as the primary model for this investigation into the measurement of students’ science identities.

Building upon Marcia’s (1966) definition of identity formation and established identity statuses, Was and Isaacson (2008) proposed the notion of an academic identity, which they deemed as constituting a special portion of Erickson’s (1959) “ego identity,” thereby agreeing that it was a distinctive component of an individual’s identity development. They postulated four academic identity statuses in congruence with Marcia’s statuses: Achieved, Foreclosed, Moratorium, and Diffused.

Specifically, an Achieved academic identity status signified an adolescent’s commitment to a set or series of academic values that are formed after a period of exploration. The Foreclosed academic identity status defined an adolescent whose commitment to their academic values was derived from influential people in their lives, but they have not yet personalized/explored this. The Moratorium academic identity status defined a period of time during which the adolescent was experiencing academic uncertainty and attempted to draw conclusions regarding their academic goals and values. Finally, the Diffused academic identity status referred to an adolescent who experienced failure in Exploration and Commitment (Was & Isaacson, 2008; Was et al., 2009). On the premise of these four statuses, Was and colleagues (2009) developed the Academic Identity Status Measure (AIM).

While identity, in general, has been extensively studied over the past 70 years and academic identity has peaked researchers’ interest over the last decade, research regarding science identity is currently gaining traction as is the need to accurately measure the construct within students (Chemers et al., 2011; Fraser et al., 2014; Hazari et al., 2018; Hill et al., 2018; Pugh et al., 2010; Robinson et al., 2018; Robnett et al., 2018; Skinner et al., 2017; Syed et al., 2018; Vincent-Ruz & Schunn, 2018; White et al., 2019; Williams et al., 2018). The first qualitative studies regarding science identity were conducted around 20 years ago (Brickhouse & Potter, 2001; Brickhouse et al., 2000; Eisenhart & Finkel, 1998; Hughes, 2001; Tan & Calabrese Barton, 2007). A common operationalization of science identity is built around Gee’s (2000) definition of identity generally as the “kind of person” one is recognized as “being” in any given context, either by oneself or with others. Gee, a linguist, attempted to provide a bridge from the study of identity to education. Carlone and Johnson (2007) employed a grounded theory approach, which led to the development of three interrelated “dimensions” of science identity: Competence, Performance, and Recognition (Carlone & Johnson, 2007). The work completed by Gee (2000) and Carlone and Johnson (2007) are commonly referenced in the literature on science identity. Indeed, these works largely reflect portions of a student’s commitment to science, but are void in investigating students’ exploration of meaningful alternatives to science or science pursuit.

Our systematic literature review of quantitative instruments used to measure science identity (Lockhart, 2021) revealed that this particular identity domain is ill-defined, not operationalized at the high school level and lacking in a theoretical framework reflective of Erickson and Marcia’s work (Chemers et al., 2011; Fraser et al., 2014; Hazari et al., 2018; Hill et al., 2018; Pugh et al., 2010; Robinson et al., 2018; Robnett et al., 2018; Skinner et al., 2017; Syed et al., 2018; Vincent-Ruz & Schunn, 2018; Williams et al., 2018). In other words, none of the instruments reviewed were founded upon the traditional identity processes of Exploration (exploration in-breadth) and Commitment. Other theoretical frameworks that were frequently utilized included: social identity theory or social cognitive identity theory (Hill et al., 2018; Merolla et al., 2012; Piatt et al, 2019), self-determination theory (Skinner et al., 2017; Williams et al., 2018), expectancy-value theory (Robinson et al., 2018), or Carlone and Johnson’s (2007) grounded theory of science identity (Fraser et al., 2014; Hill et al., 2018; Syed et al., 2018). This is a distinctive break in the literature from other identity domains, such as academic identity, that have carried forward the work of Erickson (1959) and Marcia (1966). Without theoretical frameworks based upon the traditional process of Exploration and Commitment, we are hindered from the possibility of effectively classifying individuals into one of four theoretically based science identity statuses. This, in turn, hinders uncovering the factors that uniquely influence the cultivation of science identity in students within and across these different statuses.

Furthermore, our review (Lockhart, 2021) found that only one of the instruments measuring science identity actually provided a specific definition of the construct (Skinner et al., 2017). Noting the importance of the science identity construct and its powerful predictive nature to STEM pursuit, a clear and concise definition of science identity for the target population is imperative.

Finally, several of the instruments reviewed explored science identity with a brief measure reflective of Gee’s (2000) proposition of the student’s view of themself as being a science “kind of person,” or their science self-concept (Chemers et al., 2011; Hazari et al., 2013; Hill et al., 2018; Robnett et al., 2018; Vincent-Ruz & Schunn, 2018). This brevity, though sometimes useful, inhibits our ability to more fully understand the science identity construct and its development within students.

Without a more integrated, extensive, and theoretically sound measure of science identity, we are hindered from understanding the underlying mechanisms of how and why high school students choose a STEM-related major. The goal of this study, therefore, was to provide preliminary results of a valid and reliable instrument for measuring a high school student’s science identity.

In developing the instrument, we followed Crocker and Algina’s (2008) recommendations, which consist of two main parts: (a) a qualitative part, where a preliminary measure is developed along with input from both an expert panel and a focus group; and (b) a quantitative part, which includes a pilot study with data collection and statistical analysis of the reliability and validity of the science identity (SciID) measure. Our working definition of science identity built from Marcia’s definitions of Exploration and Commitment was given as follows: “A student’s science identity is the measure to which that student has experienced a time of exploration of alternatives to science or science pursuit, and has decisively chosen to commit themselves to science by making relatively firm choices about science and engaging in activities geared towards the implementation of those choices.” Below we present these two main parts as two sequentially connected studies: Study 1 (the qualitative study) followed by Study 2 (the quantitative study).

## Study 1: qualitative study

### Methods

Through an examination of the literature on identity theory combined with the grounded theory research on science identity provided by Carlone and Johnson (2007), it was determined that science identity formation should mimic the formation of the underlying ego identity as applied to a specific domain. Thus, the science identity formation consists of two primary dimensions: Exploration (exploration in-breadth) and Commitment.

Given that no true measure of science identity existed that was foundationally based upon this traditional identity theory, an original item bank was developed to accurately reflect the dimensions of Exploration and Commitment. The SciID Scale was measured on a 5-point Likert scale ranging from “Strongly Disagree” (1) to “Strongly Agree” (5).

### Initial scale items

Noting that Exploration (exploration in-breadth) was defined by Marcia (1966) as someone having experienced a period of engagement in choosing amongst meaningful alternatives, the Exploration dimension of the SciID Scale measured the degree to which a student has undergone a period of investigation and choices among meaningful alternatives to science. Since “meaningful alternatives to science” is a broad base that can include different school subjects, hobby/interests, collegiate interests and career interests, this scale was more general in nature. A series of 14 items was initially developed to represent a student’s standing on the Exploration dimension. These items ranged from questions about a student’s level of exploration of activities and subjects in high school to their exploration of college majors (or certificates) and even careers.

The second process, Commitment, was defined by Marcia (1966) as “the degree of personal investment the individual exhibits” (p. 551). Thus, the SciID Scale measured a student’s Commitment to science based on the degree of personal investment to science that they exhibited and, therefore, was specific in nature to science. The Commitment Scale originally included 20 items developed to represent the five aspects of Competence (20%), Self-Recognition (30%), Others-Recognition (15%), Performance (20%) and Path (15%). Each question reflected a student’s degree of personal investment in science as expressed through the framework of each of these aspects.

### Revisions based on input from expert panel and focus group

An expert panel was convened that included three members: A STEM curriculum specialist (Ph.D.), a master of science high school teacher (M.S.), and a high school science teacher/science department head (B.S.). Consenting panel members received a $100 gift card for their work. Panelists were asked to (a) discuss the definitions of Exploration and Commitment provided by Marcia (1966); (b) describe in detail a student who was committed to science; (c) discuss the underlying framework of the Commitment scale and further develop/refine the five aspects; and (d) rank order the top-three and bottom-three questions per each of the Exploration and Commitment scales that most accurately/inaccurately reflected the definition of those scales. Items were thoroughly discussed and deliberated. After the conclusion of the expert panel discussion, revisions were made to the SciID Scale.

Next, a group of eight high school students, chosen based upon teacher recommendations, was convened to serve as a focus group after obtaining district approval as well as parental consent and student assent. Students received a $50 gift card for their participation. The group demographics were as follows: 25% minority, 37.5% first-generation college students, 87.5% advanced students, 75% juniors, 12.5% sophomores, and 12.5% seniors. Juniors were largely the target of the focus group as the preliminary High School Longitudinal Study of 2009 (HSLS; National Center for Education Statistics, 2015) data, which provided the framework for the study, was based upon juniors. Advanced students were included in the focus group as it was believed that these students would be more likely to demonstrate a stronger science identity and could assist in the further development/refinement of the construct.

Students were asked to engage in a descriptive analysis of each item, as they described what was understandable and relatable to the majority of high school students and what was not. Students were also asked to rank items based on their representation of the construct and relatability to high school students. Item refinement and development continued based on input from this group. Related consent and assent were gathered from all members of the qualitative study according to institutional review board human protection policies.

### Results

#### Expert panel

A set of 34 items was initially developed (14 for Exploration and 20 for Commitment). After ranking Exploration items and deliberating on their reflection of the traditional meaning of Exploration, expert panel members discussed the Commitment dimension at length and grouped together the characteristics of a student who was Committed to science. They then compared their groupings to those developed by the research team, resulting in five potential groupings of Recognition of Self, Recognition of Others, Competence, Performance, and Path, believed to reflect an aspect of a high school student’s Commitment to science. Noteworthy, four of these groupings also reflected Carlone and Johnson’s (2007) revised dimensions of science identity (Competence, Performance, Recognition of Self, Recognition of Others).

As a result of the review, three of the Exploration questions and five of the Commitment questions were refined in an effort to clarify their specific meaning. An additional three items were developed for the Commitment scale to represent a student’s interest in current events and real-life uses of science as it was believed that this was an important component to their level of Commitment. One item was recommended for deletion but was retained for focus group review, resulting in 37 items for the focus group to deliberate.

#### Focus group

Focus group members were convened to complete the extensive survey, which also included external measures. Student behavior was monitored during the survey so as to identify problematic questions. The eight high school students who formed the focus group recommended the deletion of three items on the SciID Scale due to wording problems. One of these items had also been recommended for deletion by the expert panel. Each of these three items were deleted. Further revisions of wording were made to several questions in an attempt to ensure they more accurately reflected a high school student’s interpretation of those questions.

After the conclusion of the expert panel and focus group, 14 Exploration items and 20 Commitment items resulted. Though the number of items was equivalent to what was initially developed by the research team, the item set differed from the original version. These 34 items were used for the pilot study.

## Study 2: quantitative study

### Methods

#### Participants

Students from a rural school district in southeast Texas were recruited during the spring of 2020 to participate in the study. Approximately 38% of district students were “at risk,” with 57% of the student body being economically disadvantaged. In terms of racial composition, the district population was approximately 49% Caucasian, 38% Hispanic, and 10% African American with almost equivalent majority–minority proportions.

Due to the rise of COVID-19 concerns, all pilot study measures were performed via electronic means. With the help of the school administerial staff, all the eligible high school students (*N* = 450) from the district were provided an opportunity to participate in the online SciID Scale survey through Qualtrics. An advertisement email and “Remind” texts with a link to the survey were distributed to students by the school administerial staff. To proceed to the actual SciID Scale, students had to complete a series of consenting measures; students were allowed to withdraw from the study at any time. Students who successfully completed the survey received a $10 e-gift card for their participation. All consenting and assenting measures were conducted in compliance with institutional review board human protection policies.

After cleaning the data, 156 usable surveys were retained with only one survey having any missing data. The following represents the demographics of the retained students: 63% female, 58% Caucasian, 46% economically disadvantaged, 54% Pre-AP or AP, 38% first-generation college students. Approximately 25% of students represented each grade level (9–12th).

Due to the novelty of the COVID-19 pandemic, the survey remained open for 1 month, allowing ample opportunity for participation as students were just beginning to adjust to online courses. Students were blocked from ballot-stuffing and not allowed more than one entry, but they were allowed a 7-day period of time to return to their saved surveys to complete them. Student progress was recorded.

#### Measures

In addition to the preliminary SciID Scale, which contained 34 items from the qualitative study, several other measures were collected during this phase of study, including a STEM career interest survey, as well as measures of science achievement and science self-concept. Detailed information about each scale is presented below.

*STEM career interest survey (STEM-CIS)* The STEM-CIS (Kier et al., 2014) was used to measure changes in students’ interest in STEM subjects and careers. It is based upon social cognitive career theory with subscales in science, technology, engineering, and mathematics. Rated on a 5-point Likert scale, the 44-item survey was tested with more than 1000 students who primarily resided in rural, high-poverty districts in the southeastern U.S. Confirmatory factor analyses indicated that the STEM-CIS is a strong, single-factor instrument and has four strong, discipline-specific subscales, allowing for the science, technology, engineering, and mathematics subscales to be administered separately or together. The science subscale with 11 items was used for convergent validity purposes with the Commitment dimension of the SciID Scale.

*Science achievement* Research regarding academic identity has noted significant correlations between academic identity status and academic achievement (Was et al., 2009). Moreover, the predictive nature of the different academic identity statuses for academic achievement has been well documented (Fearon, 2012; Hejazi et al., 2012; Klimstra et al., 2012; Lounsbury et al., 2005; Was & Isaacson, 2008; Was et al., 2009). It seemed sensible to conjecture that students’ science identity status, or even more simply their level of science commitment, would be correlated to their science achievement and/or predictive of their science achievement. Thus, students’ science achievement was measured as a weighted variable based upon their academic success in science and the rigor of the science courses they pursued. The variable was measured on an 11-point scale, where scores of 0–9 represent their average science grades (9 points equivalent to 95 or higher, 8 points equivalent to 90–94, 7 points equivalent to 85–89, and so on); a 2-point increase was given to those in advanced science courses. Thus, a score of 11 represented a student averaging scores of 95 + in advanced science courses. Science Commitment was expected to be a positive, significant predictor of science achievement.

*Science self-concept* Some researchers have suggested the equivalency, and thus, the interchangeable nature, of the constructs of self-concept and identity (Archer et al., 2010, 2012; Was et al., 2009). Self-concept refers to one’s view of oneself, while identity refers to the degree of exploration and commitment an individual has demonstrated within particular identity domains. Gee’s (2000) definition of identity applied to educational domains as being a “kind of person” has contributed to this equating. Moreover, several studies alluding to science identity based their operationalization of science identity on Gee’s theory, viewing this construct as being students’ view of themselves as a “science kind of person” (Hill et al., 2018; National Center for Education Statistics, 2015; Skinner et al., 2017).

In our review of this operationalization, it was determined that this science self-concept reflected students’ “recognition of themselves” as being a science person. Thus, it constitutes a portion of their Commitment to science and mimics Carlone and Johnson’s (2007) self-recognition aspect of science identity. However, differences were expected to exist between students’ commitment to science and their science self-concept; thus, a student’s science identity would not be equivalent to their science self-concept. Science self-concept was measured by the item “I view myself as a science person” within the Commitment dimension of the SciID Scale. This single item is in conjunction with previous one-item measures of science self-concept often equated to science identity (e.g., Chang et al., 2020).

#### Data analytic plan

Items of the SciID Scale were initially reviewed based upon descriptive statistics. Stata 16 (StataCorp, 2019) was used to evaluate descriptive statistics and correlational studies. Mplus 8.4 (Muthén & Muthén, 1998–2020) was used for both exploratory factor analysis (EFA) and structural equation modeling (SEM). Finally, maximum likelihood robust (MLR) estimation method was used for appropriate analyses due to the slight non-normality of a few items.

EFA was implemented to investigate the internal structure of the SciID Scale. Though research regarding (science) identity has pointed to a two-dimensional construct, no true research regarding the dimensionality of science identity under Erickson (1959) and Marcia’s (1966) theoretical framework had been conducted. Thus, it was important to explore the factor structure of the construct. Acknowledging the likely covariance between the extracted factors, the Geomin oblique rotation method was used. A scree plot was examined for initial consideration of factor retention. Determination of factor structure (i.e., which items loaded on a particular factor) was mainly based on the test of significance of the factor loadings (with *a* = 0.05). A major advantage of conducting EFA under the SEM framework in Mplus is that the overall model chi-square test as well as all the commonly used model fit statistics, including the Root Mean Square Error of Approximation (RMSEA), Standardized Root Mean Residual (SRMR) and Comparative Fit Index (CFI), are produced and may be used to evaluate the factor solution. Respective values less than 0.08 for RMSEA and SRMR and greater than 0.90 for CFI indicate an adequate model fit (Hu & Bentler, 1999). Confirmatory factor analysis (CFA) under the SEM framework was then used for the dimensionality test and model validation with external measures, including STEM-CIS (science subscale), science achievement, and science self-concept. The same model fit statistics and corresponding criteria were applied for model evaluation. The reliability of each dimension of the SciID Scale was also calculated using Cronbach’s alpha.

Vocational identity research has supported the notion of age-graded increases in vocational maturity along with changes to structures of vocational interests throughout childhood (Hartung et al., 2005). Older children demonstrate more differentiated vocational interest profiles as they become more aware of their likes and dislikes (Hartung et al., 2005). Though science identity and vocational identity were not believed to be equivalent constructs, it was reasonable to conjecture that those students who demonstrated “stronger” science identities would also demonstrate greater interests in vocational science careers. To investigate this, a composite score of the STEM-CIS (science subscale) was produced based upon the 11 items. The corresponding measurement error of the composite variable was taken into account by the reliability-adjusted method (Hsiao et al., 2018), in which the composite score was regressed on the underlying latent factor, Science Career Interest Latent Factor, while the error variance was fixed to the product of the observed score variance (0.56), and one minus the sample reliability (1–0.8713). A strong, positive relationship was expected between the Science Career Interest Latent Factor and the latent factors of the SciID Scale. The divergent validity of the SciID Scale was tested by comparing the magnitude of the path coefficients between science self-concept and the corresponding SciID latent factor. More details of this test are presented in the “Results” section.

### Results

Descriptive statistics were analyzed for each of the 34 questions on the 156 surveys. Three Exploration items were immediately identified as having excessive non-normality, resulting from high means and low variability. These items were also rated poorly by the expert panel and focus group and, therefore, were removed. Thus, 31 items remained for analyses (see Appendix A). Descriptive statistics of the remaining 31 items are provided in Table 1.

**Table 1:** Descriptive statistics for the 31 items rated on a 5-point Likert scale (*n* = 156)

Variable	Mean	SD	Skewness	Kurtosis
V1	3.37	1.52	− 0.52	1.79
V2	4.35	0.91	− 1.62	5.53
V3	3.74	1.19	− 0.57	2.26
V4	4.21	0.99	− 1.35	4.63
V5	3.87	1.14	− 0.58	2.20
V6	3.70	1.31	− 0.76	2.41
V7	3.53	1.41	− 0.53	1.92
V8	4.15	1.04	− 1.18	3.76
V9	4.15	1.02	− 1.14	3.60
V10	3.08	1.48	− 0.08	1.59
V11	3.99	1.24	− 1.22	3.50
V12	3.92	1.11	− 0.91	3.05
V13	3.53	1.12	− 0.60	2.73
V14	3.67	1.13	− 0.81	3.07
V15	3.58	1.05	− 0.68	3.12
V16	3.74	1.05	− 0.61	2.93
V17	3.16	1.40	− 0.19	1.79
V18	4.18	0.82	− 0.84	3.63
V19	3.72	1.11	− 0.59	2.58
V20	3.85	1.07	− 0.98	3.56
V21	3.19	1.24	− 0.17	2.06
V22	2.89	1.16	0.04	2.25
V23	3.49	1.09	− 0.52	2.67
V24	3.83	1.07	− 0.93	3.49
V25	2.04	1.32	1.04	2.85
V26	3.54	1.35	− 0.62	2.18
V27	3.44	1.18	− 0.67	2.65
V28	3.31	1.17	− 0.31	2.23
V29	3.74	1.16	− 0.62	2.50
V30	2.93	1.44	− 0.01	1.70
V31	3.32	1.34	− 0.32	1.94

A sample correlation matrix was then observed (see Appendix B). Furthermore, the Bartlett Test of Sphericity (with *p* < 0.001) and Kayser–Meyer–Olkin Measure of Sampling Adequacy (KMO = 0.870) indicated sufficient evidence to pursue identification of the underlying factor structure.

An EFA was conducted on the 31 items with a range of two to six factors. Two factors were chosen to be a minimum as a reflection of the two-dimensional Exploration and Commitment model. Six factors were selected to be a maximum as the Commitment dimension had five groupings of items that could potentially load onto five different factors. Initial results yielded all but one variable that loaded significantly onto one of the two hypothesized factors. However, the model fit was mediocre based on the fit statistics [${X}^{2}(298)=724.58$, *p* < 0.001, RMSEA = 0.10, CFI = 0.76, and SRMR = 0.07]. The scree plot further suggested that two strong factors were underlying the data with large eigenvalues greater than 3.5 resulting before the elbow of the graph. However, there was a clustering of four factors after the elbow of the graph with eigenvalues between 1.0 and 2.0 (see Appendix C). This gave reason to believe that there was a strong underlying two-factor solution that might be disrupted by some poorly worded items. Thus, all the items were re-evaluated.

Upon re-examination of items, it was discovered that three of the Exploration items were written in the present tense (e.g., “I don’t like to spend time thinking about my future.”), while the remaining eight items were written in the past tense (e.g., “I have thought about what major (or certificate) I want to pursue in college.”). This was deemed problematic. Thus, these three present tense items along with the item that had an insignificant loading were removed. A total of seven items remained for evaluation of Exploration.

For the evaluation of Commitment, four items were initially deemed as problematic due to poor fit and significant cross-loadings. These items were deleted. It was also discovered that one of the items was subjective in nature and yielded poor discrimination (e.g., “I work hard in my science class.”). Several other items had meanings similar to one another (e.g., “I enjoy learning about current events that involve science.” “I like seeing how science is used in the real world.”). For these, it was decided to retain only one of the items. The decision on which item to retain was based upon mean, variance, interpretability, and ranking by expert panel and focus group members. This led to the deletion of five items. Furthermore, expert panel and focus group members previously had noted an item, “I like to participate in conversations/discussions that involve science topics,” as being potentially problematic as it might not accurately reflect a high school student’s commitment to science. Their belief was that some high school students who were scientifically oriented might be shy and, therefore, not participate actively in discussions. Since other items reflected that particular aspect of science Commitment, this item was also deleted. After this evaluation, a total of nine items remained for the Commitment dimension. Thus, 16 total SciID Scale items remained for evaluation (see Table 2).

**Table 2:** SciID Scale 16 items

Dimension	Item number	Item
Exploration	V2	I have thought about what I want to do after high school
	V4	I have thought about what major (or certificate) I want to pursue in college
	V6	I have researched different college majors (or certificates) online
	V7	I have talked with someone about a college major (or certificate) that I am interested in
	V9	I have researched different careers online
	V10	I have talked with a professional in a career I am interested in about what they do in their job
	V11	I have asked someone what they think of me pursuing a particular career
Commitment	V14	My friends ask me to help them with their science homework
	V16	My parents think I am good at science
	V17	Other people expect me to pursue some type of science career (ex: healthcare, forensics, ecologist, environmentalist, computer science, meteorology, veterinarian, chemist, chemical engineer, biologist, etc.)
	V19	I want to learn more about science
	V22	I view myself as a science person
	V23	I enjoy learning about current events that involve science
	V25	I am involved in an extracurricular science activity
	V29	I will use some form of science in my future career
	V31	Science will be a part of my future after high school

With KMO = 0.883 and Bartlett’s Test of Sphericity *p* value < 0.001, another set of EFAs was conducted with the revised scale and the number of extracted factors ranging from one to three factors. The scree plot based on the revised scale suggested a strong two-factor solution with no disruption. Compared with other factor solutions, the two-factor model showed a better fit [${X}^{2}(89)=142.33$, *p* < 0.001, RMSEA = 0.06, CFI = 0.93, and SRMR = 0.05] with all significant factor loadings for each item on their hypothesized factor and a significant factor correlation of 0.40. Furthermore, the non-significant Chi-square difference test (*p* = 0.08) between the two-factor and three-factor models yielded evidence in support of the two-factor model.

The retained SciID Scale now had seven items representing the Exploration factor and nine items representing the Commitment factor. Cronbach’s alpha was calculated as 0.78 and 0.88 for the Exploration and Commitment factors, respectively. To further check the discriminant validity of the two factors, we analyzed the model under the SEM framework and constrained the correlation between the two factors to 1.0 and compared this constraint model with the original two-factor model without the correlation constraint. The statistically significant chi-square difference test with the Satorra–Bentler correction [Δχ^2^(1) = 49.50; *p* < 0.001] indicated that the two factors were not perfectly correlated and hence were two different constructs even though they were related to each other.

SEM was further used to evaluate the strength of the hypothesized relationship between a students’ Commitment to science and their Science Career Interest (SCI) and Science Achievement (Sci Ach). SCI was measured by students’ composite scores on the 11 observed variables of the science subscale of the STEM-CIS and regressed on the underlying latent factor, Science Career Interest Latent Factor (SCI-LF). Sci-Ach was measured as a weighted variable representing students’ academic success in science and the rigor of the science courses they pursued. First, all standardized factor loadings per SciID variables on their appropriate factor were significant (*p* < 0.001). Furthermore, all *R*^2^ values were significant, with *p* < 0.01 for the Exploration factor and *p* < 0.001 for the Commitment factor, suggesting that for each observed variable a significant amount of its variance was explained by its underlying latent factor (ranged from 0.22 to 0.61).

**Fig. 1:**
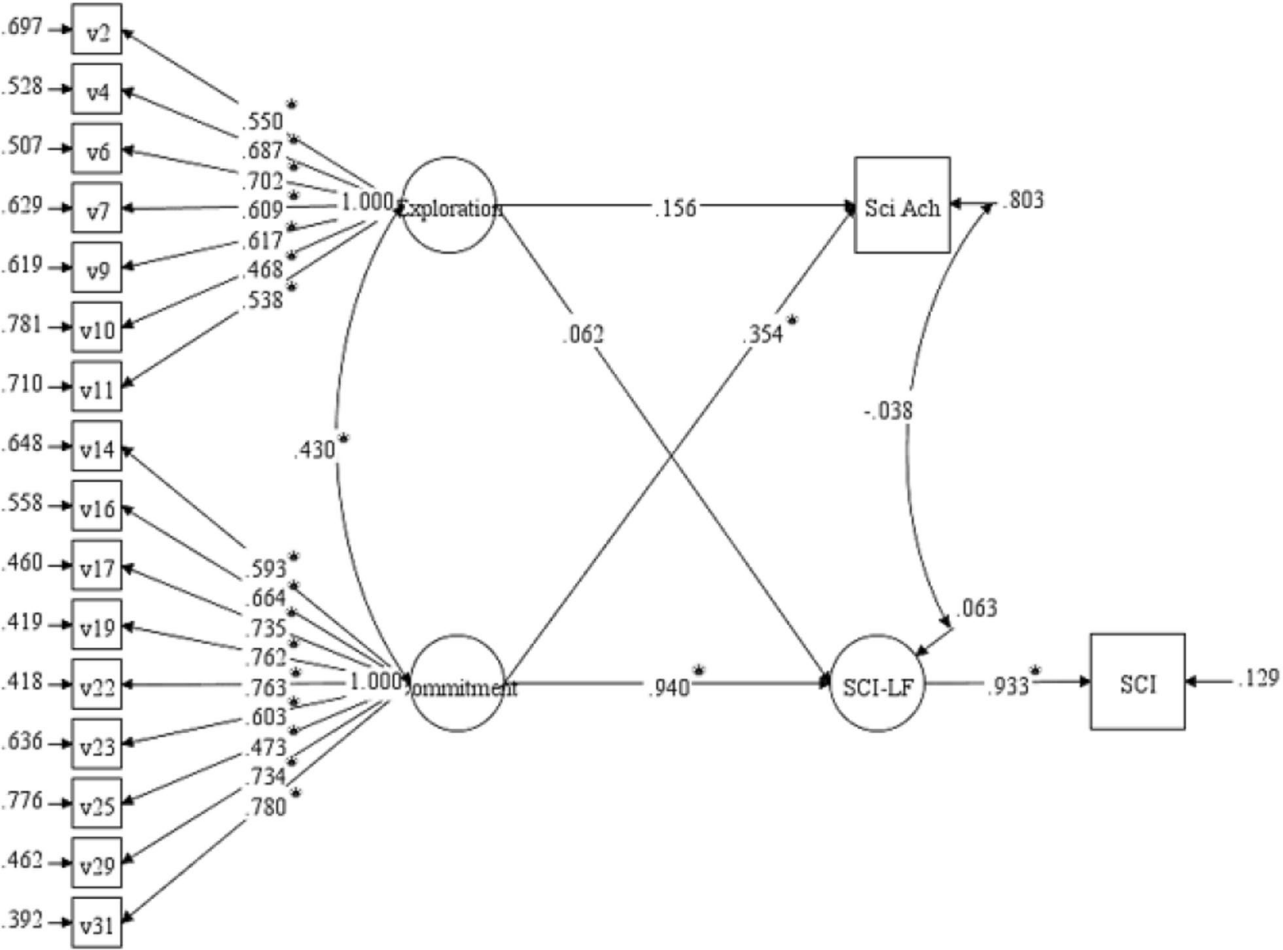
SEM investigating the convergent validity of science identity with science achievement and science career interest. *p value < .05. Results are standardized. Sci Ach = Science Achievement (measured as a weighted variable based upon student academic success in science and the rigor of the science courses the student pursued); SCI-LF = Science Career Interest Latent Factor; SCI = Science Career Interest (measured as a composite score of the Science Subscale from the STEM-CIS)

The model confirming the relationship between science Commitment and the external measures of Science Career Interest Latent Factor (SCI-LF) and Science Achievement (Sci Ach) is provided in Fig. 1. Adequate global fit statistics for the model were obtained [${X}^{2}(133)=202.80$, *p* < 0.001, RMSEA = 0.06, CFI = 0.93, and SRMR = 0.06]. Results indicated strong evidence in support of the positive, predictive nature of the Commitment factor of the SciID Scale to students’ Science Career Interest Latent Factor and Science Achievement. Though the Exploration factor was also included in this analysis, its paths to Science Career Interest Latent Factor (*p* = 0.308) and Science Achievement (*p* = 0.136) were not significant. This is largely due to the dominance of the Commitment factor. A similar model using only Exploration to predict Science Career Interest Latent Factor and Science Achievement yielded significant paths to both external variables (*p*s < 0.001), confirming that Exploration is indeed an important component of Science Identity.

**Fig. 2:**
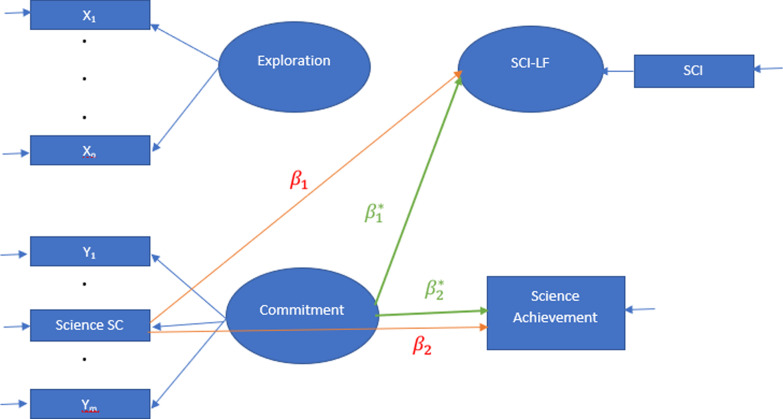
Theoretical SEM illustrating the evaluation of equivalency for science self-concept and science identity. Science SC = Science Self-Concept (“I view myself as a science person.”); Science Achievement—measured as a weighted variable based upon student academic success in science and the rigor of the science courses the student pursued); SCI-LF = Science Career Interest Latent Factor; SCI = Science Career Interest (measured as a composite score of the Science Subscale from the STEM-CIS)

**Fig. 3:**
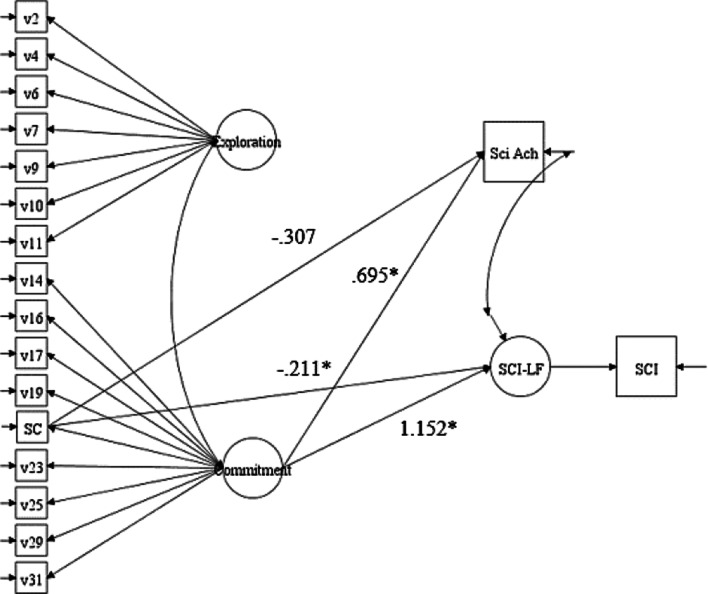
Unconstrained SEM for testing the divergent validity of science identity and science self-concept. *p < 0.05. All path coefficients are standardized. Science SC = Science Self-Concept (“I view myself as a science person.”); Sci Ach = Science Achievement (measured as a weighted variable based upon student academic success in science and the rigor of the science courses the student pursued); SCI-LF = Science Career Interest Latent Factor; SCI = Science Career Interest (measured as a composite score of the Science Subscale from the STEM-CIS)

For testing the divergent validity of Science Identity compared with Science Self-Concept, we used the variable “I view myself as a science person” and the Commitment factor as predictors to the external variables. Path coefficients between these predictors and the external variables of Science Career Interest Latent Factor and Science Achievement were first freely estimated (see Fig. 2); the corresponding coefficients were then constrained to be equal (e.g., β_1_ = β_1_^*^) in the second model. A Chi-square difference test with the Satorra–Bentler correction was performed to determine whether science self-concept and the latent factors of the SciID Scale had the same predictive power. The unconstrained model (shown in Fig. 3) with standardized path coefficients was compared to the model, where the paths from Science Self-Concept (SC) to the two external variables were constrained to equal the corresponding paths from Commitment to the same two external variables. A Satorra–Bentler corrected chi-square difference test was calculated; the significant test result [Δχ^2^(2) = 68.46, *p* < 0.001] indicated that the constrained model was too restrictive; thus, the relations between students’ science self-concept and the two external variables were not equivalent to (and actually weaker than) the same relations between the SciID Commitment factor and, hence, their science identity, and the two external variables.

We further examined the additional contribution of Commitment to the external variables compared to Science Self-Concept by computing the R-squared change (i.e., the unique additional explained variance by Commitment after accounting for the explained variance by the Science Self-Concept). A baseline model constraining the paths from Commitment to both external variables (Science Achievement and Science Career Interest Latent Factor) to zero was first estimated, and *R*^2^ values for these two external variables were observed (*R*^2^ = 0.060, and *R*^2^ = 0.495, respectively). Next, the *R*^2^ values for Science Achievement and Science Career Interest Latent Factor for the unconstrained model were observed (*R*^2^ = 0.238 and *R*^2^ = 0.985, respectively) with both being significant (*p* < 0.01). This led to *R*^2^ changes of 0.178 (or 17.8% of variance) for Science Achievement and 0.490 (or 49% of the variance) for Science Career Interest Latent Factor, which could be explained by or attributed to Commitment. In other words, students’ science identity was a significantly better predictor than science self-concept (with substantial larger explained variance) for both their science achievement and their science career interest.

## Discussion

The purpose of this study was to develop and validate a sound instrument that accurately measured a high school student’s science identity. In an effort to fulfill this purpose, the following research questions were addressed:

1. What is the dimension (latent structure) of science identity?
2. Is the newly developed SciID Scale a valid and reliable instrument?

The study sought to broaden and strengthen the research base regarding science identity and, indeed, the SciID Scale was found to show promise of being a valid and reliable instrument. Given the lack of a current science identity instrument based upon the traditional identity theory of Erickson (1959) and Marcia (1966) and the lack of focus on operationalizing the construct for high school students, it was imperative that the SciID Scale be rooted in traditional identity theory. Established in this traditional identity theory, science identity was believed to be a two-dimensional construct, reflecting the interplay between Exploration and Commitment. Through a series of factor analyses and scale revisions, this hypothesis was confirmed. Exploratory factor analysis revealed a superior two-factor model with two discriminant, though correlated, factors: Exploration and Commitment.

Through the application of SEM, the SciID Scale showed convergent validity with students’ STEM career interest and science achievement, as hypothesized. Moreover, divergent validity was established between students’ science self-concept and their science identity. Specifically, the Commitment factor demonstrated superiority in its predictive nature to STEM career interest compared to the often-utilized science self-concept that has often been equated to science identity. Thus, the measurement of students’ science identity yields an even greater predictive ability with regard to their STEM career interest than simply their science self-concept. These are not equivalent constructs. Science identity, thus, warrants much attention. Being able to accurately measure a student’s science identity should provide us with a gateway into understanding the development and stability of this construct within students and over time. Then, we can more purposefully align our interventions to effectively cultivate students’ science identities.

With good internal consistency measures for the Exploration and Commitment dimensions and the substantiation of convergent and divergent validity, it is believed that the SciID Scale is indeed a valid and reliable instrument.

### Implications for future research

The findings from this study have several implications for future research regarding science identity. First and foremost, the development of a valid and reliable instrument to measure high school students’ science identity paves the way toward understanding the developmental process and the cultivation of this type of identity within students. A larger field-test of the instrument is needed to investigate its measurement invariance, along with conducting a latent class analysis to determine if the four hypothesized identity statuses of Achieved, Foreclosed, Moratorium, and Diffused emerge. Assuming this optimal solution emerges, this creates a tremendous amount of research capabilities regarding science identity. Specifically, the accurate classification of students within science identity statuses allows for a thorough investigation into science identity development including the following questions, among others:

- What events have led students into these statuses?
- How do these statuses differ in relation to external variables, such as science-related and general academic achievements?
- What is the stability of these classifications over time?
- What predictive relationship do these statuses have with STEM career pursuit?
- Do women and minorities constitute greater proportions of certain classes?

### Limitations

An important limitation of this study pertains to the time when the pilot study was conducted. Since the pilot study took place during the beginning of the COVID-19 pandemic, some questions on the Commitment portion of the scale may have received heightened responses due to the centrality of the pandemic at the time. For instance, the item “I enjoy learning about current events that involve science.” might reflect a higher average student response than if the survey had been administered before the pandemic. However, it is difficult to know how the pandemic will shape our world for the future. Thus, this question and others of a similar nature need to be monitored over time to gain a more accurate view of actual student response.

Continuing with the impact of the pandemic, all pilot study measures were conducted via electronic means. This might also have introduced bias into the study as some students were unable to connect to the survey electronically. Though attempts were made to ensure that students of all ethnic and racial backgrounds, all SES levels, and all academic achievement levels completed the survey, that was not entirely feasible. A much larger study is needed that can help to reduce some of the potential bias introduced into this research due to its electronic nature.

Finally, the pilot study was conducted within one rural school district. A larger data set collected from a pool of diverse districts, including both urban and rural, need to be collected and analyzed to further confirm the validity and reliability of the instrument for high school students.

## Conclusions

The call for reform in STEM education remains urgent, and the COVID-19 pandemic has made it even more dire. Prior to the pandemic, employment in STEM-related occupations was projected to grow by an estimated 8.9% by 2024 (Noonan, 2017). Given the focus on science and related fields in the worldwide efforts to fight the COVID-19, one can only conjecture what those numbers will be in the future. Alarmingly, however, the STEM pipeline remains unstable. Given that a high school student’s “science identity” is the single best predictor of their pursuit of a STEM degree, it is imperative that a valid, reliable, and measurement-invariant instrument be created that accurately assesses this construct. Although a larger field-test is needed, preliminary results indicate that the SciID Scale is a valid and reliable instrument that accurately measures a high school student’s standing on this construct. The soundness of this instrument will enable policy makers and practitioners to design more effective intervention programs aimed at cultivating high school students’ science identity. The culmination of this effort will serve to increase the future STEM workforce and reduce the current leak in the STEM pipeline.

## Data Availability

The datasets generated and/or analyzed during the current study are not publicly available due their necessity and use in ongoing research efforts. However, they may be made available from the corresponding author on reasonable request.

## Appendix A

SciID scale original 31 items

**Table:**

Dimension	Item #	Item
Exploration	V1	I have chosen to stop participating in at least one extra-curricular activity that I used to be involved in
	*V2	I have thought about what I want to do after high school
	V3	I don't like to spend time thinking about my future
	*V4	I have thought about what major (or certificate) I want to pursue in college
	V5	I don’t like to think about college
	*V6	I have researched different college majors (or certificates) online
	*V7	I have talked with someone about a college major (or certificate) that I am interested in
	V8	I don’t like to think about what future jobs I want to have
	*V9	I have researched different careers online
	*V10	I have talked with a professional in a career that I am interested in about what they do in their job
	*V11	I have asked someone what they think of me pursuing a particular career
Commitment	V12	When my science class homework gets hard, I stop trying
	V13	I like to ask “why” questions in my science classes
	*V14	My friends ask me to help them with their science homework
	V15	My friends think I am good at science
	*V16	My parents think I am good at science
	*V17	Other people expect me to pursue some type of science career (ex: healthcare, forensics, ecologist, environmentalist, computer science, meteorology, veterinarian, Chemist, Chemical Engineer, Biologist, etc…)
	V18	I work hard in my science classes
	*V19	I want to learn more about science
	V20	I enjoy science
	V21	I liked science when I was younger, but I don't enjoy it as much now
	*V22	I view myself as a science person
	*V23	I enjoy learning about current events that involve science
	*V24	I like seeing how science is used in the real world
	*V25	I am involved in an extra-curricular science activity
	V26	I have a hobby that uses science (building things, investigating things, taking nature walks, learning about flowers/trees/plants, caring for animals, helping at a healthcare facility, etc…)
	V27	I can explain science concepts in a way that my friends understand
	V28	I like to participate in conversations/discussions that involve science topics
	*V29	I will use some form of science in my future career
	V30	I plan to get a science degree or certificate in college
	*V31	Science will be a part of my future after high school

*Item retained for the final 16 item instrument.

## Appendix B

Correlation Matrix of 31 Original Items

See Appendix A for a description of each variable (item).

## Appendix C

Scree plot of the original 31 items

## Abbreviations

STEM: Science, technology, engineering, and mathematics
SciID Scale: Science identity scale
STEM-CIS: STEM career interest survey
EFA: Exploratory factor analysis
MLR: Maximum likelihood robust estimation method
SEM: Structural equation modeling
RMSEA: Root mean square error of approximation
SRMR: Standardized root mean residual
CFI: Comparative fit index
CFA: Confirmatory factor analysis
KMO: Kayser–Meyer–Olkin measure of sampling adequacy
SCI: Science career interest
Sci Ach: Science achievement
SCI-LF: Science career interest latent factor
SC: Science self-concept
